# Risky sexual behaviour and human immunodeficiency virus (HIV) and acquired immune deficiency syndrome (AIDS) among healthcare workers

**DOI:** 10.4102/sajhivmed.v19i1.744

**Published:** 2018-01-26

**Authors:** Natasha Khamisa, Maboe Mokgobi

**Affiliations:** 1 Department of Public Health, Monash South Africa, South Africa; 2 Department of Psychology, Monash South Africa, South Africa

## Abstract

**Background:**

South Africa is known to have one of the highest prevalence rates of human immunodeficiency virus (HIV) and acquired immune deficiency syndrome (AIDS) globally, with one in seven healthcare workers being HIV-positive. An HIV-positive healthcare workforce is less equipped to respond to the increasing spread of the epidemic.

**Objectives:**

Assessment of the factors contributing to high HIV prevalence rates among healthcare workers is important in planning the development of human resources. This review sought to identify and understand predominant risky sexual behaviours among healthcare workers in HIV and AIDS-affected countries.

**Methods:**

This study reviewed articles focusing on sexual behaviour among healthcare workers. Major health science databases (e.g. ProQuest, Cochrane, PubMed and CINAHL) were searched for combinations of keywords including ‘healthcare workers’, ‘risky sexual behaviour’ and ‘HIV and AIDS’. Articles from a range of countries met inclusion and exclusion criteria.

**Results:**

Findings of the study revealed three main contributing factors: unprotected sex, multiple sex partners and sexual violence. Sexual violence emerged as the dominant risk factor in the majority of the studies. Most research was conducted in developed countries where the HIV infection rate is much lower than it is in developing countries.

**Conclusion:**

More research needs to be conducted in developing countries and appropriate strategies should be implemented to reduce sexual violence among healthcare workers. Appropriate procedures on reporting sexual violence coupled with education on HIV and AIDS as well as influencing attitudes and belief systems could assist in reducing the spread of HIV and AIDS within the healthcare workforce while minimising the effect on patient care.

## Introduction

Human immunodeficiency virus (HIV) and acquired immune deficiency syndrome (AIDS) are considered to be a global crisis, with healthcare systems being at the core of response efforts. However, an HIV-positive healthcare workforce is less equipped to respond to the increasing spread of the epidemic in affected countries.^1^ South Africa is known to have one of the highest prevalence rates of HIV and AIDS globally, with 12.6% of the population being HIV-positive.^2^ With HIV prevalence increasing from 4.02 million in 2002 to 6.19 million in 2015, there has been an impact on supply and demand of healthcare workers.^2^ This is likely to be exacerbated by the HIV prevalence among healthcare workers in South Africa, whereby limited research shows that among a sample of healthcare workers in South Africa, 11% are reported to be HIV-positive. Additional research reports that one in seven nurses is HIV-positive.^3^ In Zambia, research among a sample of healthcare workers confirms that up to 30% are lost annually to HIV and AIDS.^4^ It is also reported that high HIV prevalence among healthcare workers affects attrition, absenteeism and productivity, increasing costs and strain within the health sector.^5^ Assessment of risky sexual behaviours among healthcare workers is important in maintaining necessary human resources in HIV and AIDS-affected countries.

Studies have reported associations between risky sexual behaviour and HIV infection. One such study reported significant associations between inconsistent condom use and HIV infection (adjusted odds ratio [AOR] 1.41; 95% confidence interval [CI] 1.04–1.90). There was an association between increase in the number of sexual partners and HIV infection. Men reporting more than four partners were 4–12 times more likely to become infected with HIV and women reporting more than four partners were three to five times more likely to become infected with HIV.^6^ In another study exploring risk perception and HIV, participants were 17 times more likely to test HIV-positive when their perception of risky sexual behaviour was associated with multiple types of sex partners, sex preceded by alcohol use and unprotected sex (odds ratio [OR] 17.14; 95% CI 3.28–89.72, *p* < 0.001).^7^ A study in eight countries with generalised HIV epidemics showed that a change in risky sexual behaviours can reduce HIV prevalence by up to 20%.^8^

Limited research implies that risky sexual behaviour is attributed to higher HIV rates among healthcare workers. One such study showed that 21% of HIV-positive healthcare workers reported having sex with non-regular partners over the period of one year. Of these, 10% engaged in sex with multiple partners.^9^ Although risky sexual behaviour is recognised as a contributing factor to HIV and AIDS among healthcare workers, occupational exposure has been more widely studied with findings suggesting that only a small percentage (10%) of HIV among healthcare workers is attributable to exposure at work.^10^ This leaves a gap in understanding about the actual contribution of risky sexual behaviours to HIV and AIDS among healthcare workers.

Sociocultural norms play a role in increasing risky sexual behaviour, but this is rarely considered in the formation of behavioural intentions. Sociocultural contexts disrupted by stressful situations are believed to incite maladaptive behaviour. Such disruption compromises cultural identities, which then affects decision-making processes, thereby increasing the likelihood of risky sexual behaviour.^11^

Sociocultural norms influence normative and attitudinal aspects of behavioural intentions by shaping dispositions in response to situational and contextual stressors. Gender norms, for instance, may influence gender power relations in smaller subcultures of a society, where members share similar cultural beliefs and values. This may trigger risky sexual behaviours (including sexual violence as means of exerting power and discipline to subordinates).^12^ Such effect on behaviour at a sociocultural level rather than on an individual level may explain why healthcare workers engage in risky sexual behaviours despite possessing knowledge about it.

The aim of this review is to identify and understand predominant risky sexual behaviours among healthcare workers in HIV and AIDS-affected countries. It is expected that risky sexual behaviour among healthcare workers can be better understood through sociocultural considerations. This is important because healthcare workers expressing risky behaviours have severe consequences for the health system, with an infected workforce being unable to respond to the increasing prevalence of HIV and AIDS among the general population.^13^ The health system relies on healthcare workers to inform patients of their HIV status, provide prevention education as well as treat and care for infected patients. This creates the perception of healthcare workers as privileged service delivery agents resulting in less attention being paid to HIV and AIDS among this population.^1^ Given the dearth of research on HIV and AIDS among healthcare workers, they are not beneficiaries of existing interventions. Identifying the risky sexual behaviours of healthcare workers will inform appropriate interventions aimed at them, thereby preventing the loss of healthcare workers to HIV and AIDS, while maintaining service delivery to patients.

## Methods

The authors searched four major health science databases (i.e. ProQuest, Cochrane, PubMed and CINAHL) for multiple combinations of keywords including ‘healthcare workers’, ‘risky sexual behaviour’ and ‘HIV and AIDS’. A further manual search of the reference lists for articles obtained from the database search was also conducted by an independent reviewer. Articles were excluded or retained based on specific inclusion and exclusion criteria. Emerging themes were then consolidated by the authors and an independent reviewer.

**FIGURE 1:**
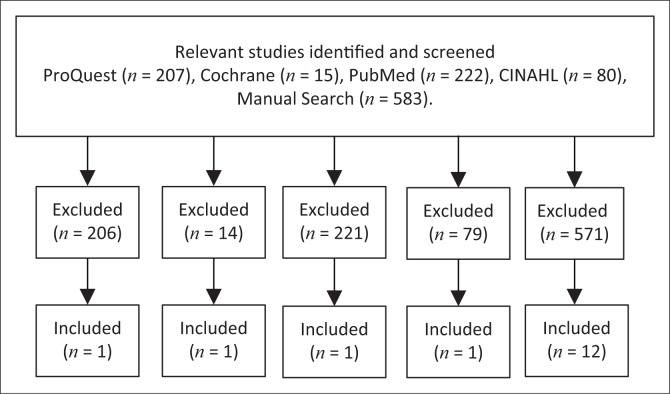
A flow chart describing the search strategy.

Inclusion criteria included full-text peer-reviewed journal articles involving quantitative studies published within the last 15 years in English. Only articles focusing on risky sexual behaviour and HIV and AIDS among samples of healthcare workers were retained. Articles published in languages other than English in conference proceedings and newspapers more than 10 years ago were excluded. Articles focusing on risky sexual behaviour of sex workers, adolescents and patients were also excluded. In addition, articles including insufficient details about the sample and context as well as articles focused on other health outcomes were excluded. This is illustrated in Figure 1.

## Results

After applying inclusion and exclusion criteria, 16 articles were retained and analysed according to country, sample and findings (Table 1).

**TABLE 1:** Country, sample and results of identified studies.

Database	Articles	Retained	Country	Findings
ProQuest	207	1	Turkey	A total of 715 nurses working in 38 hospitals were recruited. Of these, 380 nurses participated in the study. About 32.6% of nurses reported sexual violence, 94.4% of the sexual violence was perpetrated by doctors and 97.9% of nurses reported that they had not received training on coping with sexual violence. Sexual violence is more prominent than physical abuse but least reported.^14^
Cochrane	15	1	Chile	A total of 750 healthcare workers were recruited. Of these, 555 agreed to participate. Educated healthcare workers reported less unprotected sex (*B* = −0.84, *t*(524) = -3.24, *p* < 0.001) but not fewer sexual partners (*B* = −0.03, *t*(530) = −0.90, *p* < 0.05).^15^
PubMed	222	1	Canada	A total of 3802 doctors were recruited. Of these, 770 participated. Nine per cent of doctors reported sexual violence by other doctors. Although a small proportion, this was reported to have grave consequences including loss of confidence in clinical abilities and mental health issues, which could affect patient care.^16^
CINAHL	80	1	Taiwan	A total of 700 nurses were recruited for this study and 545 participated. About 12.9% of nurses experienced sexual violence by another healthcare worker especially during night shifts (OR = 2.3, 95% confidence interval = 1.29–4.16).^17^
Manual search	58	1	Burkina Faso	Of 1697 healthcare workers recruited, 1570 participated in the study. About 21% reported having had sex with non-regular partners, 10% reported having more than one sexual partner and 13% did not use condoms. Those engaging in risky sexual behaviour were more likely to be in rural areas (23.4% vs 19.3%, *p* = 0.07 for non-regular partners and 16.7% vs 7.5%, *p* = 0.02 for non-condom use).^9^
	32	1	India	Of 141 healthcare workers recruited, 135 agreed to participate. About 21% of doctors were perpetrators of sexual violence against other doctors and 11% against nurses. Only 15% of healthcare workers were aware of workplace guidelines.^18^
	58	1	Turkey	Of 1204 nurses recruited, 622 participated. About 51% reported sexual violence by other nurses and 77% by doctors. Education was associated with the likelihood of sexual violence (*X^2^* = 8.69, *p* = 0.034).^19^
	32	1	Australia	A total of 538 out of 2489 participants agreed to be part of the study. About 27% of nurses reported sexual violence by doctors and 13% by other nurses.^20^
	58	1	Thailand	A total of 545 out of 594 nurses participated in the study. About 75% reported sexual violence by other nurses.^21^
	45	1	Canada	Of 300 nurses approached, 181 participated in the study. About 30.7% of violence against nurses was sexual and 65.9% of this was inflicted by another nurse and 59.1% by a doctor.^22^
	34	1	Zambia	A total of 692 healthcare workers from five hospitals participated in the study. About 33% of healthcare workers and 24% of their partners had been tested for HIV, 26% of sexually active healthcare workers had multiple partners and 37% of these did not use condoms.^1^
	34	1	Rwanda	A total of 360 healthcare workers were recruited and 350 participated. Only 17% reported using condoms.^23^
	58	1	Turkey	A total of 1209 healthcare workers from 34 work sites were selected. About 11.2% of healthcare workers are sexually violent towards their colleagues.^24^
	58	1	USA	Participants consisted of 2166 healthcare workers from four healthcare institutions. About 9.1% participants reported sexual violence by co-workers (including doctors).^25^
	58	1	Israel	Of the 250 nurses recruited, 220 participated. About 3.8% of nurses reported sexual violence perpetrated by doctors. Only 20% – 25% of incidents are reported. About 80.9% of incidents are reported to the nurse in charge.^26^
	58	1	Taiwan	A total of 536 healthcare workers participated in this study. About 2.4% experienced sexual violence from their co-workers. Age was associated with risky sexual behaviour, with older healthcare workers being more susceptible (OR = 5.46, *p* = 0.022).^27^

Of the 16 studies included, 11 studies (69%) focusing on risky sexual behaviour among healthcare workers have been conducted in developed countries and only five studies (31%) in developing countries.

Of the 16 studies included, 12 studies identified sexual violence as one of the risky sexual behaviours among healthcare workers. Of these, eight studies confirmed that doctors are often the perpetrators of sexual violence, with only four studies showing that sexual violence was perpetrated by nurses. Eight studies showed that nurses are often victims of sexual violence.

Four studies confirmed unprotected sex as one of the risky sexual behaviours among healthcare workers. More educated healthcare workers reported less unprotected sex, whereas healthcare workers from rural areas reported more unprotected sex.

Three studies confirmed multiple sex partners as one of the risky sexual behaviours among healthcare workers. Education had no effect on the number of sexual partners with healthcare workers in rural areas having a higher number of sexual partners.

Additional details on the above-mentioned findings are included in Table 1.

## Discussion

The aim of the study was to assess factors contributing to high HIV infection rates in the healthcare workforce by reviewing studies conducted in various countries. The reviewed studies reported risk factors contributing to an increase in HIV infection rate among healthcare workers, which could be summarised under three main themes: unprotected sex, multiple sex partners and sexual violence. It is worth noting that only four studies reported unprotected sex and multiple sex partners as the main risk factors. Sexual violence emerged as the dominant risk factor in the majority of the studies and includes doctor-on-doctor sexual violence, doctor-on-nurse sexual violence or nurse-on-nurse sexual violence. The majority of sexual violence seems to be perpetrated by doctors.^14,16,18,20^

Doctors perpetrating sexual violence against other healthcare workers could be explained by power dynamics. Doctors are, by virtue of the organogram of the healthcare system, ‘superior’ to other healthcare workers who take orders or instructions from them. These power imbalances render other healthcare workers vulnerable to doctors, making them less likely to report sexual violence for fear of reprisals or victimisation by the doctors.^14^ A similar explanation of power dynamics could be proffered for doctor-on-doctor violence, where a senior doctor perpetrates sexual violence on a junior doctor. Such behaviour has been conceptualised as a natural socialisation process commonly considered a salutary rite of passage as reported in a number of studies.^28^

Sexual violence is also viewed as an issue of gender norms. The majority of healthcare workers on the lower end of the hierarchy are women, which makes them more vulnerable to sexual violence.^29^ Studies have reported that women are seven times more likely to experience sexual violence compared to men.^30^ Male sex role socialisation influencing sexual beliefs is expressed in the form of sexual violence as a means of attaining masculinity and dominance.^31^ Other factors are associated with working environments and working conditions.^32^

Any form of sexual violence has serious negative consequences, which include, but are not limited to, loss of confidence in clinical abilities and mental health issues, known to have adverse consequences on patient care.^16^ This in the context of high HIV prevalence in developing countries could diminish the healthcare workforce and place further strain on the supply and demand of healthcare workers.^3^

It is important to note that sexual violence is an unwanted and unreciprocated sexual behaviour, to which victims do not consent. Research suggests that experiencing sexual violence has been associated with an increase in risky sexual behaviours, whereby victims are more likely to have unprotected sex with multiple sex partners. This is attributed to a loss of sexual decision-making power and increases susceptibility to HIV infection.^21,23^

Studies have shown that many healthcare workers reported not using condoms regularly, despite some having multiple sex partners. It has been found that many healthcare workers believe condoms are ineffective in preventing HIV infection, with 60% confirming their beliefs about the effectiveness of condom use in preventing the spread of HIV.^1^ Such findings are attributed to a lack of knowledge about HIV and AIDS on the part of many healthcare workers, with only 46% of healthcare workers participating in a study on knowledge, attitude and practice about AIDS and condom utilisation, having adequate knowledge about AIDS.^23^ This is further supported by findings showing that more educated healthcare workers are less likely to engage in unprotected sex.^15^ Healthcare workers in rural areas report having more multiple sex partners. This has been attributed to poor social support and separation from families when rural healthcare workers move to urban areas for work.^9^

Several gaps have been identified in the literature. Most research was conducted in developed countries where the HIV infection rate is much lower than it is in developing countries. No studies were found for South Africa in particular, which is reported to be the significantly impacted by HIV and AIDS. Only a few of the studies included in this review focus on HIV and AIDS prevention among healthcare workers. The majority of the studies available mostly focus on other samples including sex workers, adolescents and patients.

### Limitations

It is acknowledged that 16 articles may limit the generalisability of findings in this review. Furthermore, with the majority of existing research focusing on risky sexual behaviours among healthcare workers in developed countries, it is difficult to address policy implications for developing countries where HIV and AIDS is more prevalent. However, given the dearth of research on risky sexual behaviours among healthcare workers, this handful of articles provides a unique insight into sexual behaviours of healthcare workers as well as related outcomes (HIV). Further research exploring risky sexual behaviours among healthcare workers is important in reducing HIV and AIDS among this population, especially in developing countries.

### Recommendations

It is recommended that research should focus more on exploring the factors contributing to high HIV rates among healthcare workers in developing countries. Appropriate implementation strategies overcoming the challenges posed by power imbalances are necessary to empower healthcare workers to make a formal complaint without experiencing stigma, discrimination or intimidation. At a national level, recognition and action are required from governments and professional regulatory bodies, while improving healthcare practice and organising community action at a local level.^33^ At organisational level, it must be ensured that complaints are taken seriously and treated fairly, despite being directed against doctors.^18^ Working environments and working conditions must be improved in order to address the context within which the problem occurs.^34^ Similar prevention strategies have been shown to reduce sexual violence in the workplace by 40%.^21^ Solutions must also be centred on challenging female vulnerability. The cognitive appraisal theory emphasises promoting women’s action on sexual violence by educating them on recognising cues associated with sexual violence, understanding psychological barriers to effective resistance and self-defence training. These types of interventions allow women to assess the situation in preparation for response.^35^

Healthcare workers should be educated on the consequences of risky sexual behaviour, including condom use especially with multiple sex partners. Such education should be targeted at both the perpetrators and victims of sexual violence.^23^ Education has been found to be an effective strategy in reducing HIV and AIDS.^36^ Interventions involving training on ethics and sexual rights as well as communication and mutual respect are important in addressing sexual violence in the workplace.^33^ Supportive working environments for rural healthcare workers in urban areas are important in addressing risky sexual behaviours associated with multiple sex partners. Studies have confirmed reduced risky sexual behaviours among people living with HIV and AIDS through support interventions within working environments as well as through social and community support.^37^

## Conclusion

HIV and AIDS remains a global crisis with South Africa bearing the brunt of its prevalence. Healthcare workers are not immune to this condition, which stifles efforts to effectively combat the disease. Sexual risk behaviours, especially sexual violence among healthcare workers, can only exacerbate the problem by increasing infection rates and compromising availability of human resources. Appropriate procedures on reporting sexual violence coupled with education on HIV and AIDS as well as influencing attitudes and belief systems could assist in reducing the spread of HIV and AIDS within the healthcare workforce while minimising the effect on patient care.
